# Missing Black males among preterm births in the US, 1995 to 2019

**DOI:** 10.1371/journal.pone.0295557

**Published:** 2024-03-18

**Authors:** Tim A. Bruckner, Suman Chakrabarti, Brenda Bustos, Ralph Catalano, Alison Gemmill, Joan A. Casey, Hedwig Lee

**Affiliations:** 1 Department of Health, Society, and Behavior, and the Center for Population, Inequality, and Policy, University of California, Irvine, Irvine, California, United States of America; 2 International Food Policy Research Institute, New Delhi, India; 3 Program in Public Health, University of California, Irvine, Irvine, California, United States of America; 4 School of Public Health, University of California, Berkeley, Berkeley, California, United States of America; 5 Department of Population, Family and Reproductive Health, Johns Hopkins Bloomberg School of Public Health, Baltimore, Maryland, United States of America; 6 Department of Environmental and Occupational Health Sciences, University of Washington School of Public Health, Seattle, Washington, United States of America; 7 Department of Sociology, Duke University, Durham, North Carolina, United States of America; Adam Mickiewicz University Faculty of Biology: Uniwersytet im Adama Mickiewicza w Poznaniu Wydzial Biologii, POLAND

## Abstract

**Background:**

In the US, non-Hispanic (NH) Black birthing persons show a two-fold greater risk of fetal death relative to NH white birthing persons. Since males more than females show a greater risk of fetal death, such loss *in utero* may affect the sex composition of live births born preterm (PTB; <37 weeks gestational age). We examine US birth data from 1995 to 2019 to determine whether the ratio of male to female preterm (i.e., PTB sex ratios) among NH Black births falls below that of NH whites and Hispanics.

**Methods:**

We acquired data on all live births in the US from January 1995 to December 2019. We arrayed 63 million live births into 293 “conception cohort” months of which 2,475,928 NH Black, 5,746,953 NH white, and 2,511,450 Hispanic infants were PTB. We used linear regression methods to identify trend and seasonal patterns in PTB sex ratios. We also examined subgroup differences in PTB sex ratios (e.g., advanced maternal ages, twin gestations, and narrower gestational age ranges).

**Results:**

The mean PTB sex ratio for NH Black births over the entire test period (1.06, 95% Confidence Interval [CI]: 1.05, 1.07) is much lower than that for NH white births (1.18, 95% CI: 1.17, 1.19). NH Black PTB sex ratios are especially low for twins and for births to mothers 35 years or older. Only NH white PTB sex ratios show a trend over the test period.

**Conclusions:**

Analysis of over 10 million PTBs reveals a persistently low male PTB frequency among NH Black conception cohorts relative to NH white cohorts. Low PTB sex ratios among NH Black births concentrate among subgroups that show an elevated risk of fetal death. PTB sex ratios may serve as an indicator of racial/ethnic and subgroup differences in fetal death, especially among male gestations.

## Introduction

An estimated 30 to 70 percent of human pregnancies end before a live birth [1, 2]. Males predominate among detectable pregnancy losses in the 2^nd^ and 3^rd^ trimesters [3]. In addition, males appear more frequent among births delivered preterm (PTB; <37 weeks gestational age at delivery) [4, 5]. Known causes of male excess among late pregnancy losses, and of live PTB, remain elusive [6].

In the US, non-Hispanic (NH) Black births show a two-fold greater risk of fetal death (i.e., ≥20 weeks) relative to non-Hispanic white births [7]. To the extent that this elevated risk of NH Black fetal death disproportionately affects male fetuses, such loss *in utero*—even pre-20 weeks—may affect the sex composition of live births born preterm. A recent study in California, for instance, estimated a large “missingness” of males among non-Hispanic Black PTB (relative to expected levels derived by comparisons to other race/ethnicities such as non-Hispanic white infants) [4]. The Authors, and previous researchers [8], suggest that high levels of “left-truncation” *in utero*, especially among gestations to NH Black birthing persons, may account for relatively fewer NH Black male PTB.

We view the ratio of male to female preterm births (i.e., PTB sex ratios) as worthy of further investigation for three reasons. First, quantifying racial/ethnic differences in PTB sex ratios using contemporary US data may provide insight into the potentially large (and difficult to measure) racial disparity of mid-to-late pregnancy losses. Previous work speculates that socioeconomic disadvantage may account for a portion of the racial disparity in maternal and fetal morbidity. An estimated 19.5% of NH Black families (vs. 8.1% of NH white families) had incomes below the federal poverty line [9]. In addition, median household income of NH Black adults (in 2016) was $39,500, as compared to $65,000 for NH white adults [10]. Second, if sex ratios vary over time in response to a variety of external stimuli, characterizing changes over time in PTB sex ratios in the US may assist with identifying prenatal exposures that adversely affect the viability of male gestations [11]. Third, elevated pregnancy loss may, through “left-truncation” [12, 13], result in lower neonatal mortality for the conception cohort that survives to live birth. Left truncation refers to selective attrition of frailer members of the pregnancy cohort before live birth, which may result in relatively fewer (but, on average, hardier) members of that cohort that are observable at live birth. To the extent that skewed PTB sex ratios may reflect left truncation in a conception cohort, such information may assist with predictions of disparities in neonatal survival among preterm deliveries.

We contribute to the literature by describing PTB sex ratios in the US over a 25-year period (1995 to 2019). We focus on PTB sex ratios among NH Black births and compare them to NH white and Hispanic births. Based on prior literature [4], we hypothesize that the mean of NH Black PTB sex ratios will fall below that of NH white PTB sex ratios. Next, we array the birth data by conception cohort and explore potential *changes over time* in PTB sex ratios. Furthermore, given our interest in exploring the extent to which the PTB sex ratio may serve as a sensitive indicator of elevated pregnancy loss, we examined specific sociodemographic subgroups (e.g., twin births, births to persons >35 years) which the literature suggests might show notable departures from the global mean of PTB sex ratios [14].

## Methods

### Data and variables

We retrieved data on all live births in the US, from January 1995 to December 2019, from natality files assembled by the National Center of Health Statistics (NCHS), Division of Vital Statistics. These files include the longest series of natality data, with individual-level variables, available to us at the time of our inquiry. We excluded birth records missing gestational age (<3% of all records) and restricted the analyses to live birth records with plausible gestational age (i.e., 20 to 45 weeks). The University of California, Irvine Committee for the Protection of Human Subjects approved this study (protocol # 20195444).

The files contain month and year of birth and gestational age (GA) in weeks (which we multiplied by 7 to yield GA in days). We used the NCHS combined (based on both obstetric estimate and date of last menstrual period) estimate of GA. The NCHS natality file also includes race/ethnicity, sex, singleton/twin status, maternal age, and maternal education, which (as described below in *Analysis*) allows subgroup examinations of our key dependent variable.

To estimate the month of conception for each live birth, we used GA and date of birth information. NCHS data provide month and year of birth but not exact birth dates. We assigned all births a random day of birth from the set of possible days of birth in that month (e.g., January = 31, February = 28 except in leap years in which February = 29). We then subtracted GA in days from the assigned birth date to estimate month of conception. This process produced a time series of 293 monthly conception cohorts, with the first “full” conception cohort occurring in August 1994 (and born in 1995) and the last “full” conception cohort occurring in December 2018 (and born in 2019).

We classified race/ethnicity of the birth according to the race/ethnicity of the birthing person recorded on the birth certificate. Our principal focus involves non-Hispanic (NH) Black births. Consistent with the literature, we use NH white births as the main comparison group. We also, however, use Hispanic births as a comparison group to further assess whether trends, or levels, in NH Black sex ratios appear distinct. We created a count variable, by race/ethnicity and sex of infant, for the overall number of conceptions in each month that produced a live birth. In addition, we created separate variables for the number of conceptions in each month that resulted in a preterm birth (PTB). We defined PTB as any live birth <37 weeks GA.

### Analysis

We focused on the sex ratio of PTB among NH Black births, which (consistent with the literature [15]) we define as the ratio of male to female PTB births. We first plotted, for 293 conception months, sex ratios of PTB for NH Black and NH white births. Second, we employed ordinary-least-squares linear regression analysis to fit a year variable (continuous, from 1 to 25, where 1994 = 1, 1995 = 2, etc.) and 11 binary calendar month variables (i.e., February through December, with January as a referent month). In this regression, for each race/ethnicity the US-conception month is the unit of analysis (n = 293). We therefore draw inference to the ecological (i.e., group) level rather than at the level of individual births. This approach assumes that the US collects birth data consistently over time such that it shows no artefactual “breaks” or “jumps” over time. Our inspection of the time-series plots, as well as a careful review of the data documentation of the US Natality file, indicates that the dataset meets these assumptions.

We summarized the intercept and year slope coefficients of the PTB sex ratio by race/ethnicity. We assessed other functional forms of the year variables, such as quadratic and cubic terms, to determine whether coefficients changed in the presence of non-linear annual trends. We performed all analyses using Scientific Computing Associates (SCA, River Forest, IL).

Although relatively low PTB sex ratios can arise from fewer than expected male PTB, low values could also arise from an increase in female PTB. For this reason, if ordinary-least-squares regression results showed a secular trend in PTB sex ratios, we then explored whether changes in male more than female births accounted for this trend. Next, to determine whether sociodemographic subgroups considered at greater risk of fetal loss show lower PTB sex ratios, we repeated the racial/ethnic-specific regression analyses by subgroup. The high-risk subgroups of interest include twins (vs. singletons), births to persons 35 years or older (vs. <35 years), and births to persons with less than a high school education (vs. ≥ high school education).

In addition, we examined the PTB sex ratio calendar month coefficients, with January as the referent coefficient and fixed at 0, to ascertain any calendar and/or seasonal patterns in PTB sex ratios. We also divided PTB sex ratios into smaller GA ranges, and performed the aggregate-level regression analyses described above, to permit comparison of results to previous literature [4]. We classified the following three GA ranges: periviable (i.e., 22 to <26 weeks), early PTB (26 to <32 weeks), and late PTB (i.e., 32 to <37 weeks). These ranges match clinically relevant PTB risk categories [5] and have a sufficient number of births to calculate sex ratios (e.g., >400 births for NH Black periviable GA for every conception month, which is the GA- racial/ethnic category of lowest frequency). We also included Hispanic births in all analyses so that readers could compare NH Black PTB sex ratios to a referent category other than only NH white births.

Data Availability Statement: We make all underlying data available in the S1 Dataset file.

## Results

**Fig 1:**
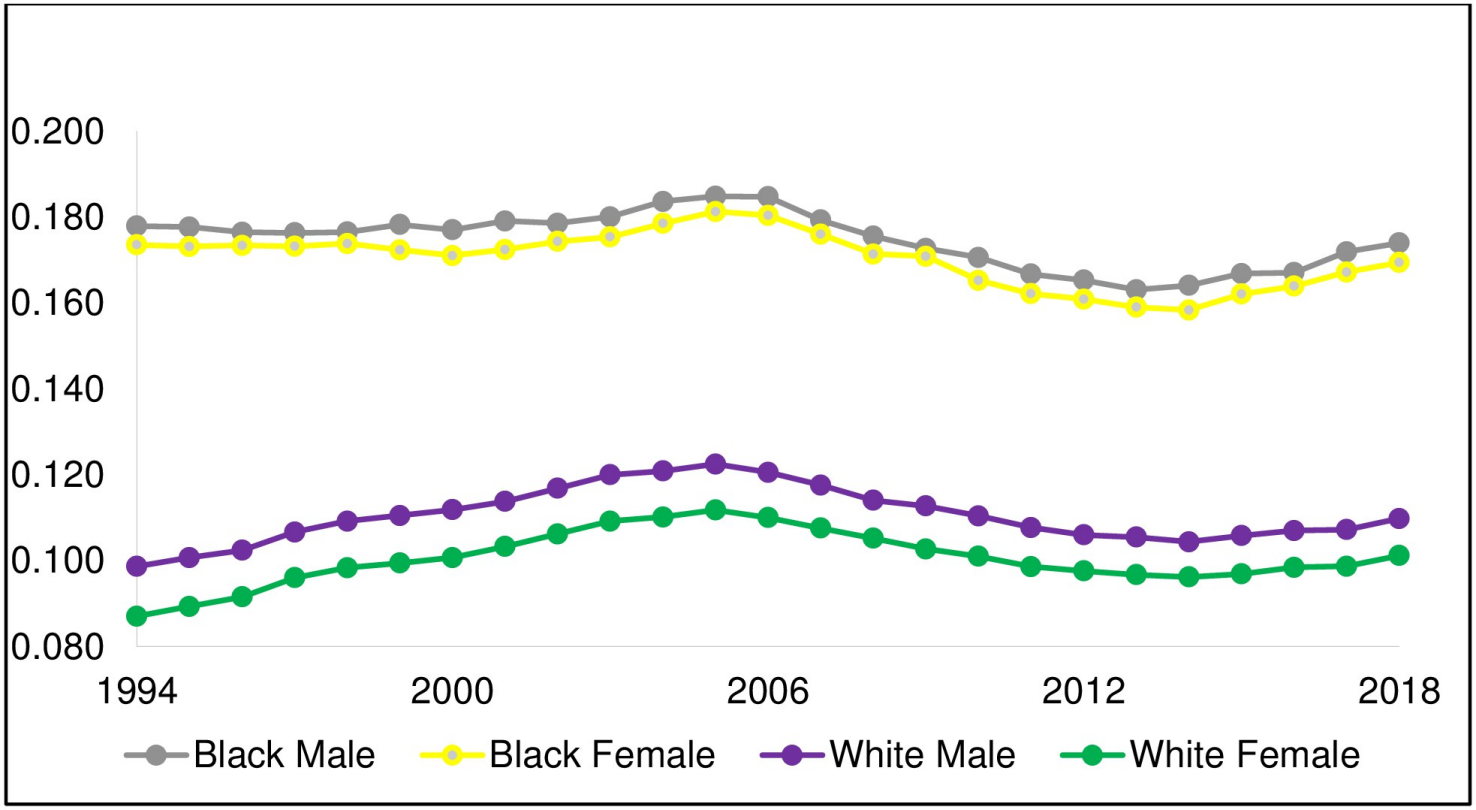
Preterm birth sex-specific incidence, by conception year, for non-Hispanic Black and non-Hispanic white birthing persons.

**Fig 2:**
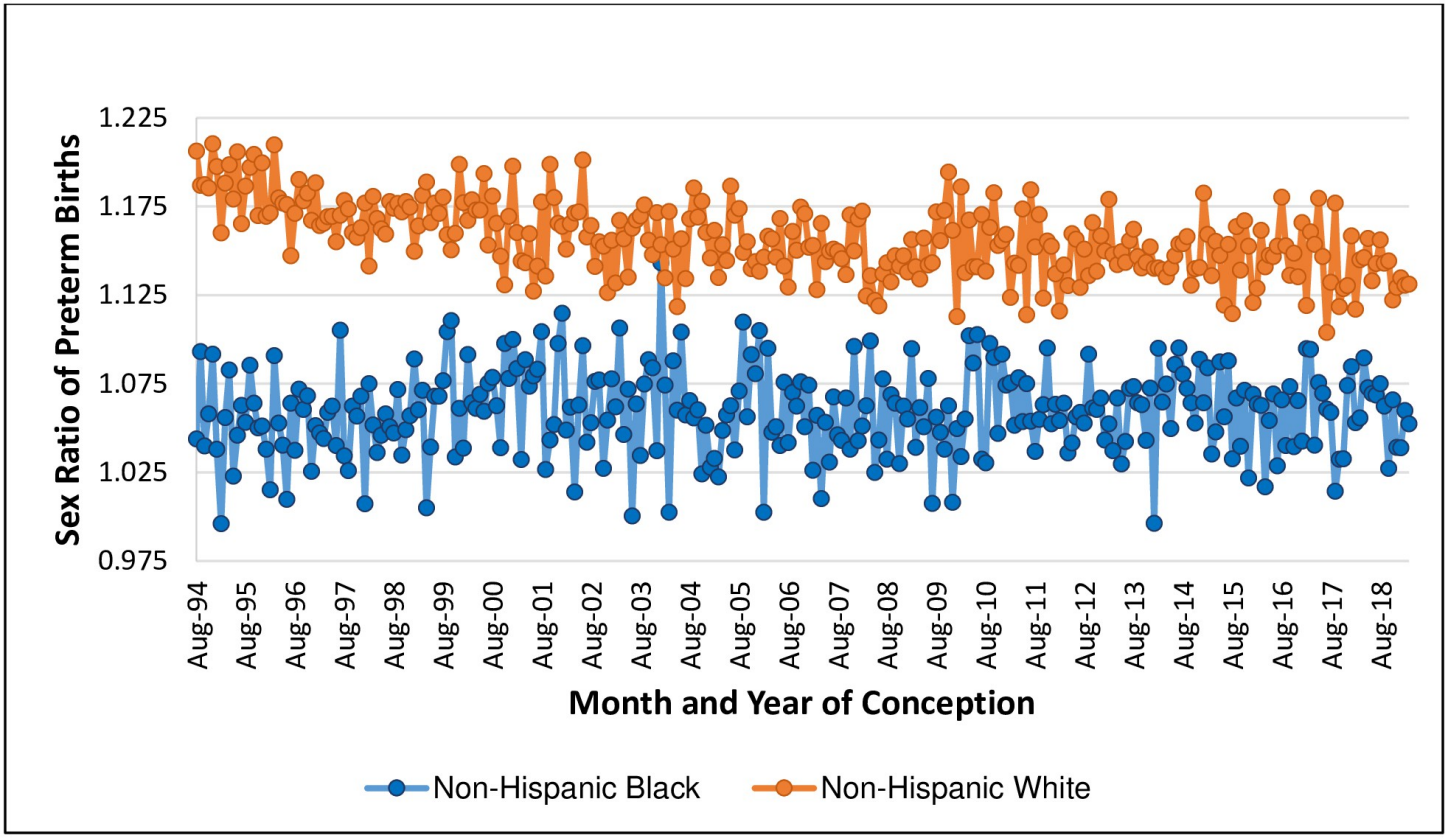
Preterm birth sex ratio, by conception month, for non-Hispanic Black and non-Hispanic white birthing persons.

**Fig 3:**
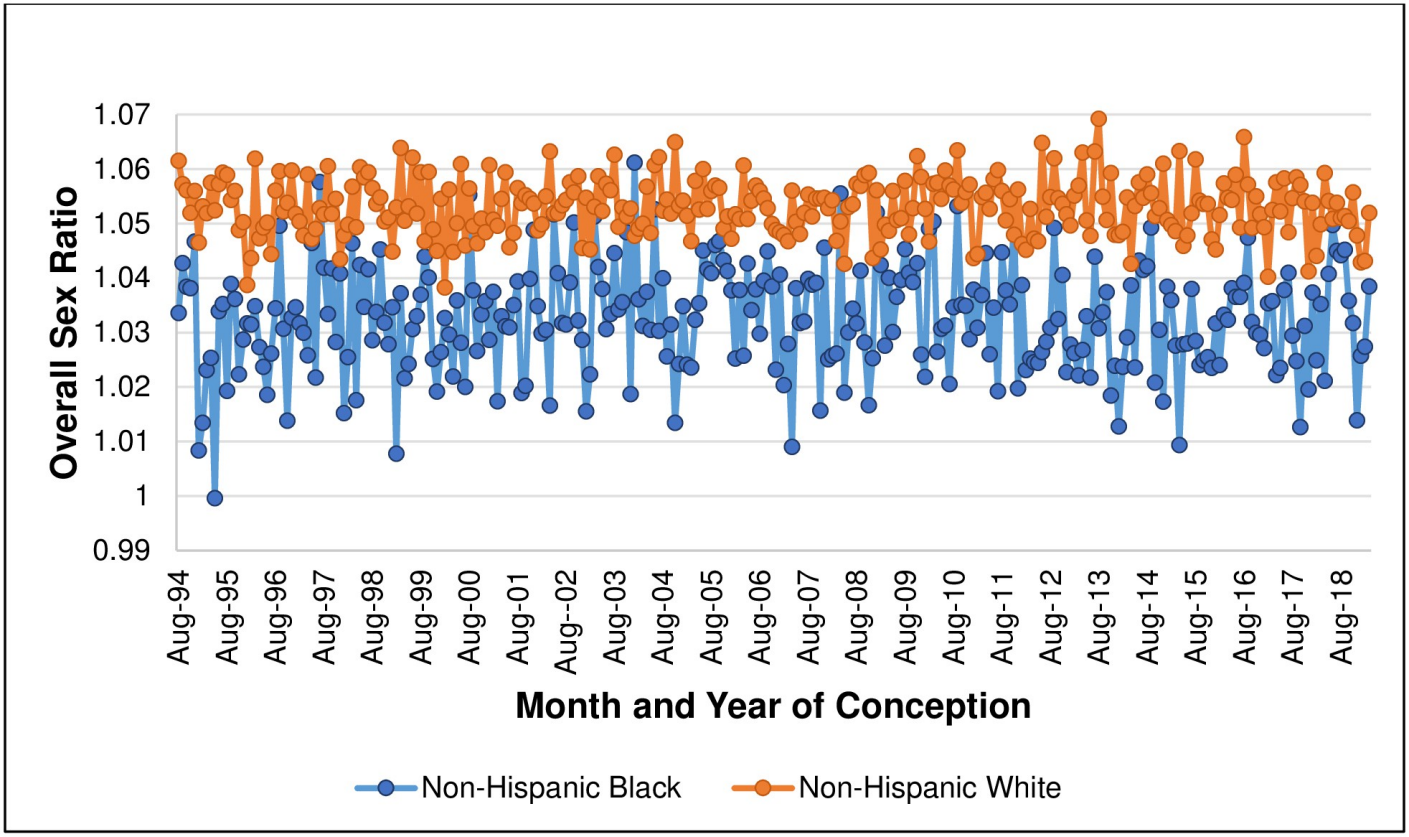
OVERALL birth sex ratio, by conception month, for non-Hispanic Black and non-Hispanic white birthing persons.

Our analysis includes 63,831,757 births over 293 conception months, of which 2,475,928 NH Black, 5,746,953 NH white, and 2,511,450 Hispanic infants are PTB. Fig 1 plots the sex-specific incidence of PTB for NH Black and NH white births (plots for Hispanic births appear in S1 and S2 Figs). Consistent with prior reports, the incidence of PTB is much greater for NH Black relative to NH white births [16]. Fig 2 plots, for 293 conception months, PTB sex ratios for NH Black and NH white births. For every month, the PTB sex ratio for NH white births is greater than that for NH Black births (mean difference: .098, range: .01 to .18). The series for NH Black births shows more variability than does the NH white series owing to a smaller conception cohort size (monthly mean = 48,970, relative to mean for NH white: 185,019). Whereas the NH Black series appears to show a stable mean, the NH white series gradually declines until the end of 2007. After 2007, the NH white series appears to show a stable mean. The secular decline in the PTB sex ratio for NH white contrasts that of the *overall* NH white sex ratio of all live births, which shows a stable mean (Fig 3).

Racial/ethnic-specific regression results (Table 1), in which we estimate a constant and year term (while controlling for calendar effects), support the general inference from the PTB sex ratio plots. Consistent with the literature, the mean PTB sex ratio for NH white births (1.18) is much greater than that for NH Black births (1.06). The year term for the NH Black PTB sex ratio shows no secular trend. By contrast, the PTB sex ratio for NH white births shows a strongly detectable annual decline over the test period (year coef: -.0016, 95% Confidence Interval [CI]: -.0018, -.0014). Inclusion of a quadratic term for NH white series modestly improves fit and indicates a flattening of the rate of decline after 2010 (S1 Table). Further inspection indicates that decreases in the numerator (i.e., male PTB) predominantly account for the secular decline in PTB sex ratios among NH whites (S2 Table). In addition, whereas the calendar month coefficients for PTB sex ratios did not indicate a consistent pattern, conceptions in summer months show elevated sex ratios among *term* births for all race/ethnicities (S3 and S4 Figs).

**Table 1:** Coefficients (standard errors) for constant and year terms in models predicting sex ratio of births^†^.

Variables	NH Black		NH White		Hispanic	
	Constant	Year	Constant	Year	Constant	Year
Sex Ratio PTB	1.06 (.0054)**	.0001 (.0002)	1.18 (.0037)**	-.0016 (.0001)**	1.175 (.0057)**	-.000023 (.0002)
Sex Ratio Periviable Birth	1.102 (.0246)**	-.0012 (.0009)	1.196 (.0261)**	-.0012 (.0010)	1.200 (.037)**	-.0024 (.0014)
Sex Ratio ePTB	1.071 (.0121)**	-.0011 (.0004)*	1.180 (.0112)**	-.0024 (.0004)**	1.18 (.0175)**	-.0011 (.0006)
Sex Ratio latePTB	1.054 (.0061)**	.0004 (.0002)*	1.180 (.0041)**	-.0015 (.0001)**	1.175 (.006)**	.0002 (.0002)
Sex Ratio term births	1.024 (.002)**	-.00009(.00008)	1.043 (.001)**	.00001 (.00004)	1.031 (.0019)**	-.0002(.00006)**
*For Preterm Births Only*						
Maternal Age						
Sex ratio <35 years	1.063 (.0059)**	.00005 (.0002)	1.186 (.0042)**	-.0016 (.0002) **	1.1776(.0063)**	.0001 (.0002)
Sex ratio 35 years+	1.041 (.014)**	.0009 (.0005)	1.164 (.008)**	-.0013 (.0003)**	1.176 (.0149)**	-.0003 (.0005)
Maternal Education						
Sex Ratio, > High School	1.054 (.0089)**	.0001 (.0003)	1.184 (.0049)**	-.0017 (.0002)**	1.185 (.0105)**	-.0004 (.0004)
Sex Ratio, HS grad or less	1.065 (.0071)**	.00004 (.0003)	1.178 (.006)**	-.0015 (.0002)**	1.176 (.0066)**	.00002 (.0002)
Plurality						
Sex Ratio, Singleton	1.070 (.0060)**	.0002 (.0002)	1.219 (.0045)**	-.0016 (.0002)**	1.189 (.0064)**	.0006 (.0002)*
Sex Ratio, Twins	0.986 (.0143)**	.0005 (.0005)	1.031 (.0085)**	-.0009 (.0003)**	1.034 (.0170)**	-.0014 (.0006)*

^†^ coefficients for calendar month not shown here (but available in the Supporting Information).

(*p < 0.05; ** p < 0.01)

**Fig 4:**
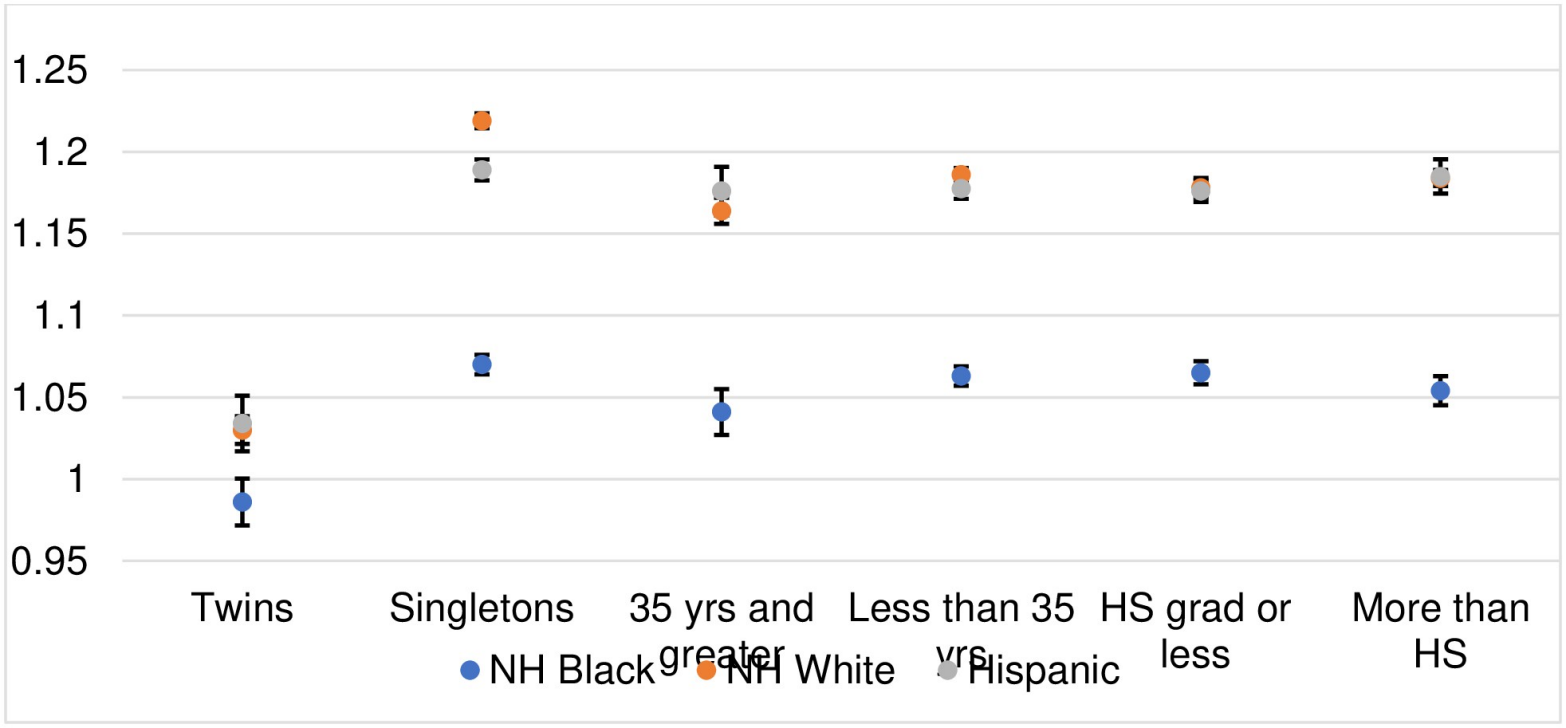
Coefficients (95% CIs) for the preterm birth sex ratio by race/ethnicity and subgroup.

Closer inspection of PTB sex ratios among subgroups at greater risk of fetal death reveals lower means (Fig 4). Older birthing persons (35+ years) show lower PTB sex ratios than do younger birthing persons (<35 years) for both NH Black and NH white groups. For NH white individuals, the 35+ year series also shows a secular decline (similar to the overall PTB sex ratio). However, among older NH Black birthing persons, the PTB sex ratio appears stable. In addition, for all race/ethnicities, PTB sex ratios of twins are much lower than that of singletons. NH Black twins show the lowest PTB sex ratio of any subgroup examined (constant: 0.986, 95% CI: 0.958, 1.014). The PTB sex ratio of twins, moreover, shows a secular decline for NH white (and Hispanic) births but not for NH Black births.

We focused on NH Black PTB sex ratios and the extent to which they diverge from the referent group of NH whites. However, for comparison purposes we also examined Hispanic PTB sex ratios in the regression framework (Table 1). Results for Hispanic births, both in terms of PTB sex ratios overall and for sociodemographic subgroups, are quite similar to that of NH whites. One notable difference involves no detected secular decline in PTB sex ratios for Hispanic births (see Appendices E and F for Hispanic plots).

**Fig 5:**
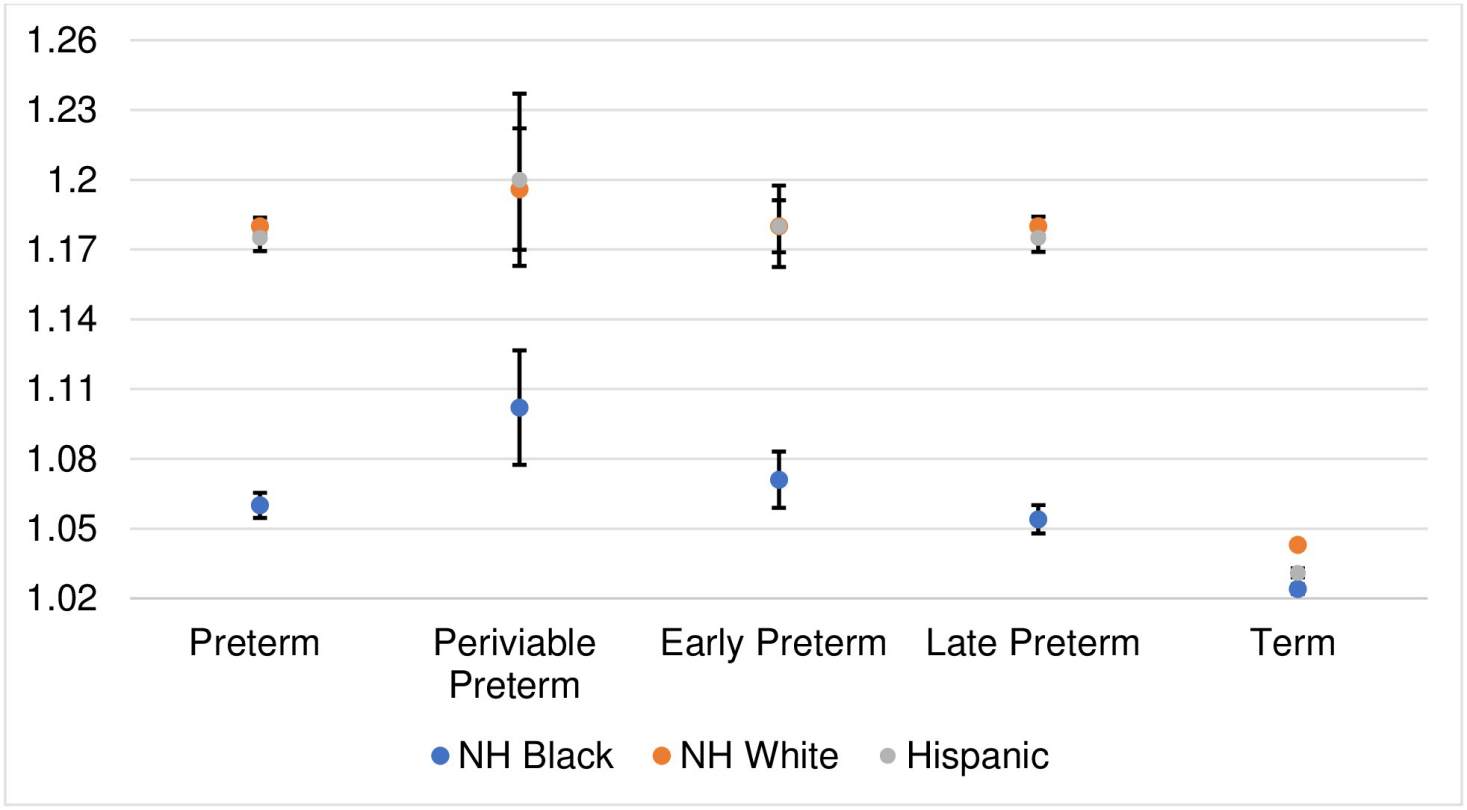
Coefficients (95% CIs) for preterm birth sex ratio by race/ethnicity and gestational preterm age ranges.

We then subdivided PTB into three gestational periods (i.e., periviable, early PTB, and late PTB) to determine whether means of sex ratios, or secular trends in sex ratios, differ across the GA range of PTB. Unlike a prior report in California [4], sex ratios in the US are largest at periviable birth and lowest at late PTB—for all race/ethnicities (Table 1; see Fig 5 for plots). The secular decline in PTB sex ratios among NH whites, moreover, appears driven by declines among early and late PTB (but not periviable PTB).

Although not central to our exploration, the observed declining trend in PTB sex ratios for NH whites compelled us to examine whether variation over time in the cohort *incidence* of PTB (Fig 1) may explain a portion of the decline in the PTB sex ratio. We therefore inserted overall PTB incidence for each of the 293 conception cohorts (i.e., aggregated across sex) for NH whites into the regression equation which estimates PTB sex ratios. We observe an inverse relation between PTB incidence and PTB sex ratios among NH whites. Inclusion of overall PTB incidence, however, does not account for the declining trend in that the year coefficient (i.e., -.0016) remains unchanged from the original test. Next, we controlled for monthly cohort measures of sociodemographic composition of PTB sex ratios (i.e., mean maternal age, percent of non-singletons, and percent of mothers with an education of high school degree or less) and re-estimated the regression equation. This specification assesses whether well-described demographic and compositional changes over time in childbearing drive the declining PTB sex ratio among NH whites. Findings, shown below, indicate for NH white PTB sex ratios that the year coefficient remains negative and statistically detectable, which suggests that compositional factors do not account for the declining trend (S3 Table). Furthermore, we disaggregated the US data into four large Census-defined regions (i.e., Northeast, Midwest, South, and West) and re-ran the regression analyses. Results (S4 Table) indicate relatively consistent PTB sex ratios for NH whites, and a declining trend in three of the four regions.

## Discussion

For reasons that remain unclear, NH Black births in the US have historically shown a relatively lower frequency of males among PTB deliveries. We used contemporary US data over a 25-year period to determine whether the PTB sex ratio (an indicator of male frequency) varies over time, and to assess whether specific subgroups at elevated risk of fetal death also show particularly low PTB sex ratios. Analysis of over 10 million PTBs from 1995 to 2019 reveals a persistently low male PTB frequency among NH Black conception cohorts relative to NH white (and Hispanic) cohorts. Particularly low PTB sex ratios among NH Black births concentrate in twins and among birthing persons over 35 years—two sociodemographic groups that show an elevated risk of fetal death [17, 18]. Given that males predominate in detected fetal losses, the left-truncation argument contends that excess male fetal loss would result in relatively fewer male live PTB. Consistent with that argument, our findings indicate that PTB sex ratios may serve as an indicator of racial/ethnic and subgroup differences in fetal death, especially among male gestations.

Our analysis makes several contributions. First, we update prior research [4] with recent data spanning a long period and document a persistently low PTB sex ratio among NH Black births. Factors related to the changing sociodemographic composition of mothers (i.e., maternal education, twinning rate, and maternal age; see S3 Table) do not account for this low PTB sex ratio. Although our exploration did not set out to identify causes of the “missing” NH Black males, speculation centers on the possibility that preeclampsia and infection may increase risk of PTB in a sex-independent manner [19]. In addition, the fact that we observed the lowest NH Black PTB sex ratios among twins and births to mothers 35 years or older coheres with the argument that that low PTB sex ratios logically follow from excess loss of males *in utero*. We intend to explore the potential left-truncation mechanisms further by collecting spontaneous loss data (where available), by sex, for these conception cohorts.

Second, we find a declining trend in PTB sex ratios among NH white (but not NH Black) births over a 25-year period. This race-specific decline broadly agrees with Davis and colleagues’ report [11]. We hesitate to make direct comparisons to prior work, however, given that they examined *overall* sex ratios and focused on a much earlier epoch (i.e., post-World War II). Third, our subgroup analyses for NH white births indicates that the steepest sex ratio decline occurs among early PTB (26 to <32 weeks) ages, which makes it unlikely that changes in obstetrical practices which increased general risk of delivery among late-preterm gestations drove the NH white results. In addition, subgroup analyses by maternal age, maternal education, and twinning status do not suggest large demographic shifts over time (e.g., advancing maternal age) as a main cause. We note that the decline in PTB sex ratios among NH whites concentrates in 1994 to 2008 cohorts. Additional research on this phenomenon may therefore want to focus on data conception cohorts pre-2009. Such an extension could take the form of examining metropolitan- or state-level changes over time in employment (by region or industrial classification code—especially in the Northeast and South, where the PTB sex ratio decline is most steep; see S4 Table), given the recent work which documents declining economic conditions of the NH white working class [20].

Another novel observation involves the discovery of the greatest male “bias” in live births at the peri-viable gestational age range, followed by early PTB, then by late PTB, and finishing with term gestational ages. This gestational-age gradient in male bias extends earlier work [5] and holds across all three race/ethnicities. This pattern appears consistent with the notion that, especially at very early gestational ages, PTB has more in common with stillbirths than they do with term births [21]. The sex ratio of periviable births, most of which would have been stillbirths before the advent of aggressive neonatal intensive care, should be further investigated regarding whether it may approximate the sex ratio of pre-20-week spontaneous abortions.

Although we intended our exploration to provide information useful to understanding disparities in preterm birth in the US, our findings also have implications for theories regarding selection *in utero* [15]. Our results appear consistent with the argument that the relatively high perinatal cost of sustaining male offspring makes the timing of their parturition critical to maternal fitness [22, 23]. Natural selection may have conserved a set of heritable mechanisms, over human history, which result in fewer than half of human conceptions surviving to birth (and with a disproportionate amount these losses being male) [1, 24]. Such mechanisms, if operating differentially in response to various ecological pressures across places and times over much of human history, could plausibly produce dramatically different “starting points” of sex ratios across contemporary populations [25, 26].

Not all results cohere with the argument that excess male fetal loss among disadvantaged mothers accounts for low PTB sex ratios. For instance, mothers with lower educational backgrounds do not show lower PTB sex ratios. This finding, as well as several reports documenting lower birth sex ratios among all births (i.e., term and preterm combined) in Black populations in Africa and the US [25–28], suggest the need to consider additional explanations. Genetic or epigenetic factors could plausibly produce different baseline expectations of PTB [29], and of PTB sex ratios, by race/ethnicity. Such inquiries into these factors would benefit from careful measurement of such factors, rather than inferring a set of genetic characteristics from socially-derived racial/ethnic classification alone [30, 31]. In addition, we encourage careful theorizing about the extent to which genetic factors could explain the large NH Black / NH white divergence of sex ratios among PTBs we report here—which is much greater than the well-documented racial/ethnic divergence of overall birth sex ratios—and is unlikely to be explained solely by genetic factors [25–28].

Strengths of our exploration include the population-based nature of the US vital statistics data which reduces stochastic variability in estimates of monthly rates. We also analyze a large time span in which risks of PTB varied substantially over time. Next, the alignment of births by conception month permits estimation of PTB sex ratios for specific cohorts and specific gestational ages that are not confounded by period effects (e.g., seasonality). We, however, focused our exploration on NH Black births and therefore did not examine other populations (e.g., Asian) who the literature reports may have skewed sex ratios [32, 33]. In addition, we did not examine regional or state variation in sex ratios. Such additional exploration may identify plausible antecedents of high or low PTB sex ratios that would warrant further refinement and testing.

Previous work in California finds that elevated unemployment coincides with an increased risk of male fetal death [34]. Whereas this finding supports a potential “economic hardship” mechanism for elevated male fetal death and lower PTB sex ratios, prior work did not focus on race/ethnicity and did not have the individual and neighborhood-level employment and income data which researchers have now linked to birth records (e.g., Kennedy-Moulton et al. [35]). This circumstance, combined with recent innovative work documenting the large Black / white employment and income gaps which persist across generations [36], should compel future work on economic antecedents of fetal loss and low PTB sex ratios. Such work would benefit from linkage of granular geographic and individual-level economic data to vital birth and fetal death records and quantify the relative contribution of economic hardship to low PTB sex ratios among NH Black births.

Demographers and epidemiologists continue to debate the extent to which cohort left-truncation of gestations affects the survival characteristics of live births [8, 37]. As this argument relates to our sex ratio findings, an unusually low level of NH Black male PTB may reflect a high level of losses *in utero* among this group that, if born live, would have resulted in a live-born PTB but which would show an elevated risk of neonatal death. Whereas we cannot test this argument with our data, future linkage of monthly conception cohorts to neonatal death records may illuminate the extent to which cohort markers of left-truncation—such as the PTB sex ratio—predict a cohort’s risk of neonatal death especially among NH Black males.

## Supporting information

S1 Fig Sex-specific incidence of preterm birth, by conception year, for Hispanic and non-Hispanic white birthing persons. (TIF)

S2 Fig Sex ratio of preterm births for Hispanic (purple) and non-Hispanic white (orange) birthing persons. (TIF)

S3 Fig Calendar month coefficients predicting sex ratio of PTB, by race/ethnicity. January fixed at 0.
(TIF)

S4 Fig Calendar month coefficients predicting sex ratio of term births, by race/ethnicity. January fixed at 0.
(TIF)

S1 Table Coefficients (95% CIs) for non-Hispanic white PTB sex ratio after adding a quadratic year term. (*p < 0.05; ** p < 0.01).
(TIF)

S2 Table Coefficients (95% CIs) for constant and year terms predicting number of sex-specific preterm live births. ^**†**^ coefficients for calendar month not shown.
(TIF)

S3 Table Coefficients (standard errors) for the constant and year term, *with inclusion of covariates*, predicting the sex ratio of live preterm births. ^**†**^ coefficients for calendar month not shown here (but available in the S3 Fig). (*p < 0.05; ** p < 0.01).
(TIF)

S4 Table Region-specific^‡^ coefficients (standard errors) for constant and year terms in linear regression models predicting the sex ratio of preterm births. ^**† ‡**^ Northeast (Connecticut, Maine, Massachusetts, New Hampshire, New Jersey, New York, Pennsylvania, Rhode Island, and Vermont); Midwest (Illinois, Indiana, Iowa, Kansas, Michigan, Minnesota, Missouri, Nebraska, North Dakota, Ohio, South Dakota, and Wisconsin); South (Alabama, Arkansas, Delaware, Florida; Georgia, Kentucky, Louisiana, Maryland, Mississippi, North Carolina, Oklahoma, South Carolina, Tennessee, Texas, Virginia, Washington, D.C., and West Virginia); West (Alaska, Arizona, California, Colorado, Hawaii, Idaho, Montana, Nevada, Oregon, New Mexico, Utah, Washington, and Wyoming). ^†^ coefficients for calendar month not shown. (*p < 0.05; ** p < 0.01).
(TIF)

S1 Dataset (XLSX)
