# Supplementary figures and images for: Missing Black males among preterm births in the US, 1995 to 2019
Source: PLoS One. 2024 Mar 18;19(3):e0295557. doi: 10.1371/journal.pone.0295557 (PMC10947666; doi:10.1371/journal.pone.0295557)

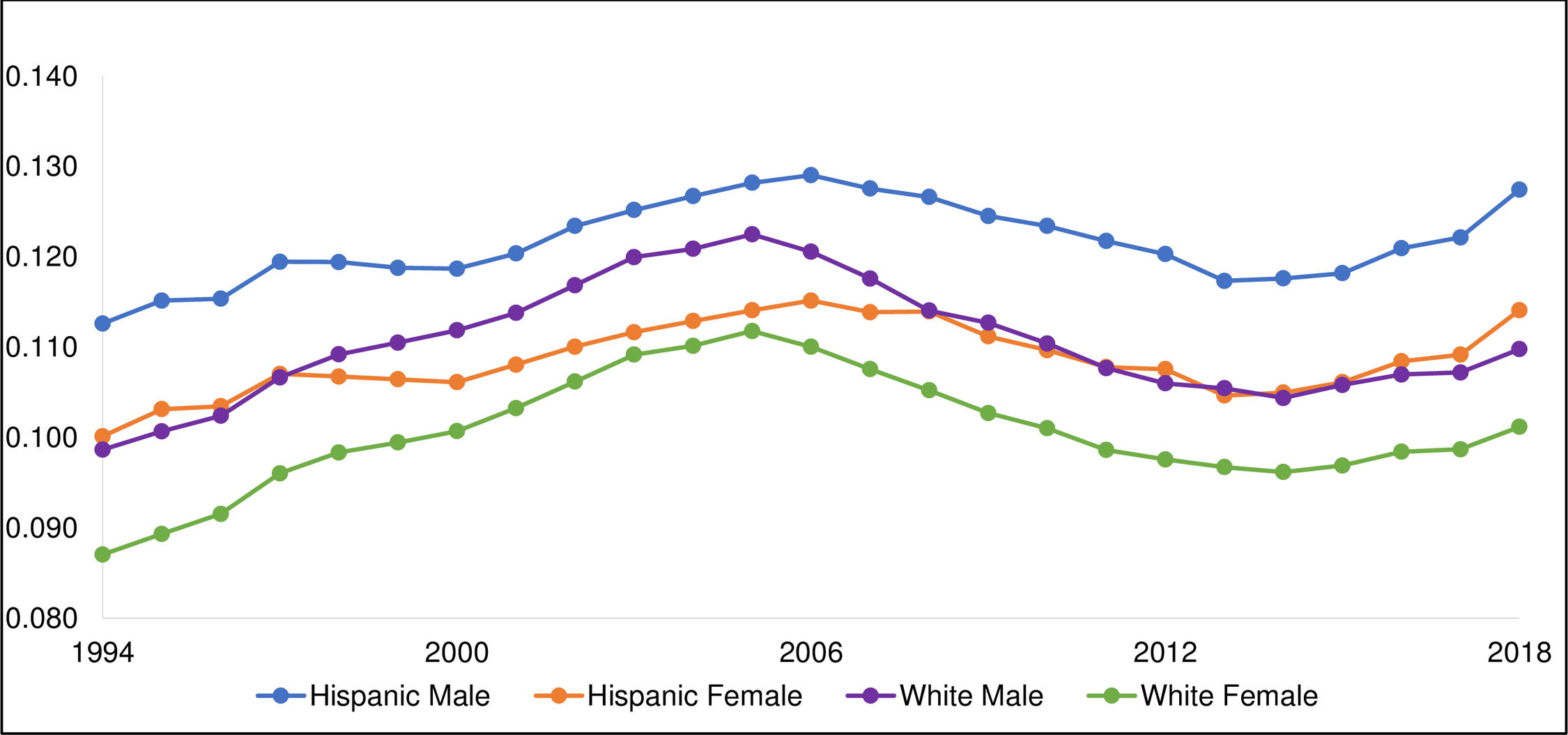

Supplement: S1 Fig — (TIF) [file pone.0295557.s001.tif]

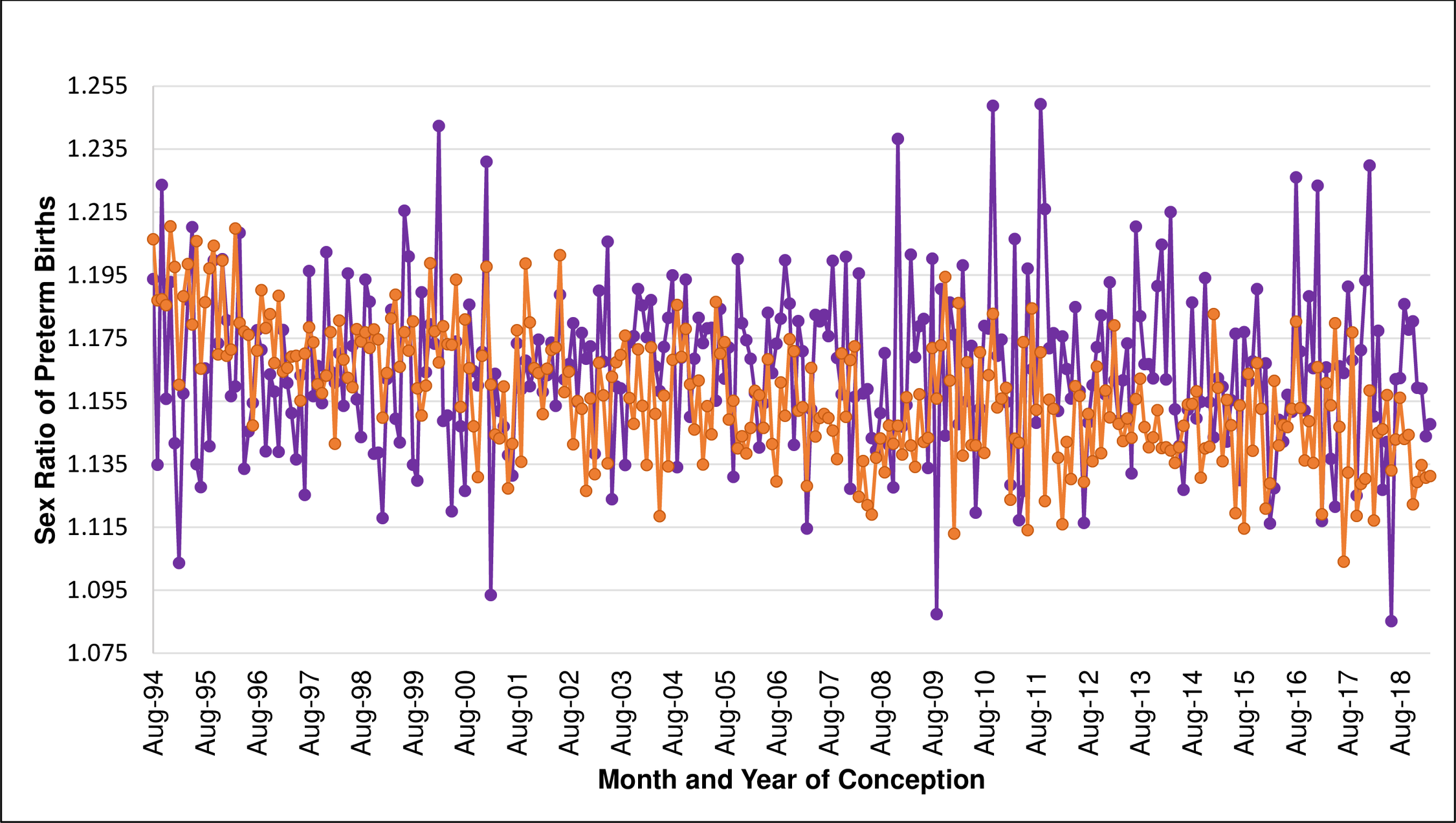

Supplement: S2 Fig — (TIF) [file pone.0295557.s002.tif]

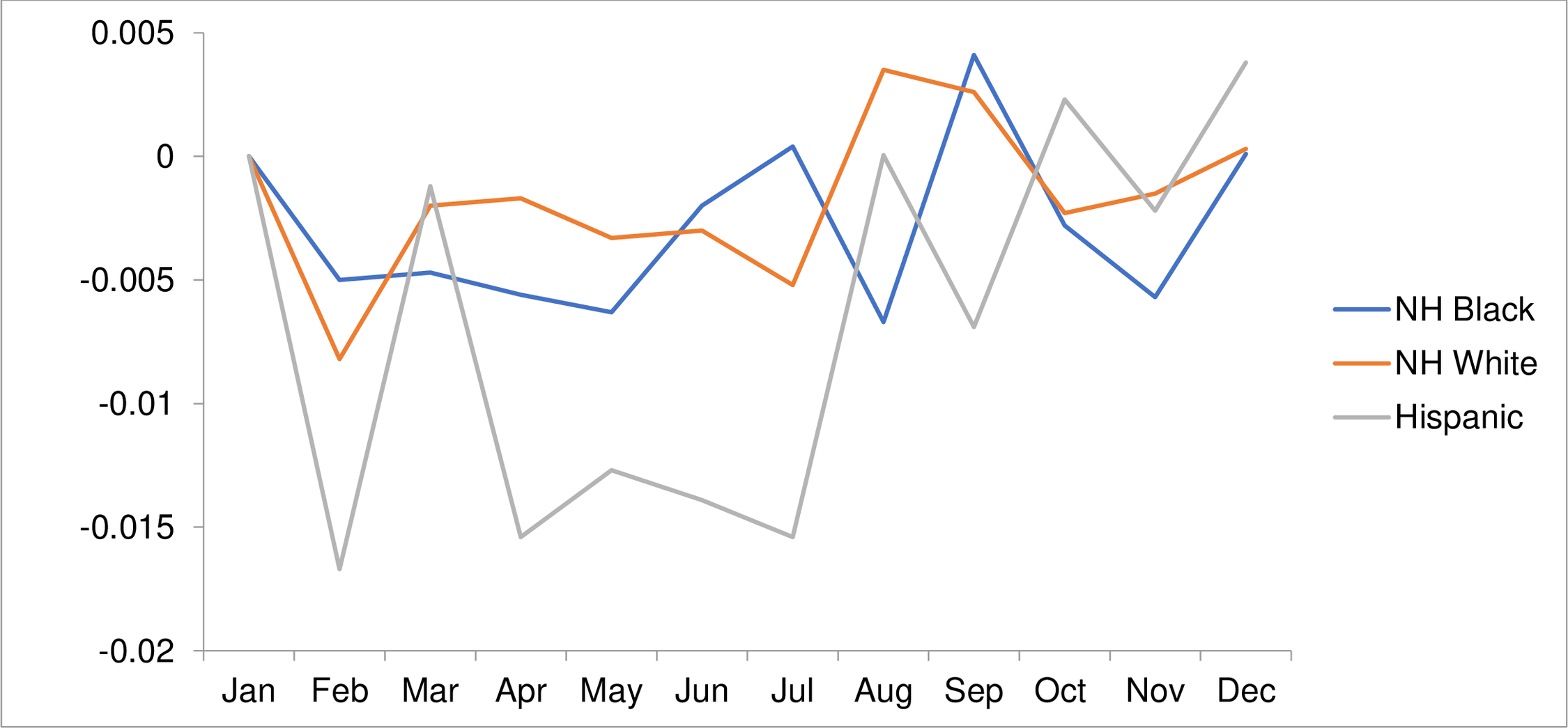

Supplement: S3 Fig — January fixed at 0. (TIF) [file pone.0295557.s003.tif]

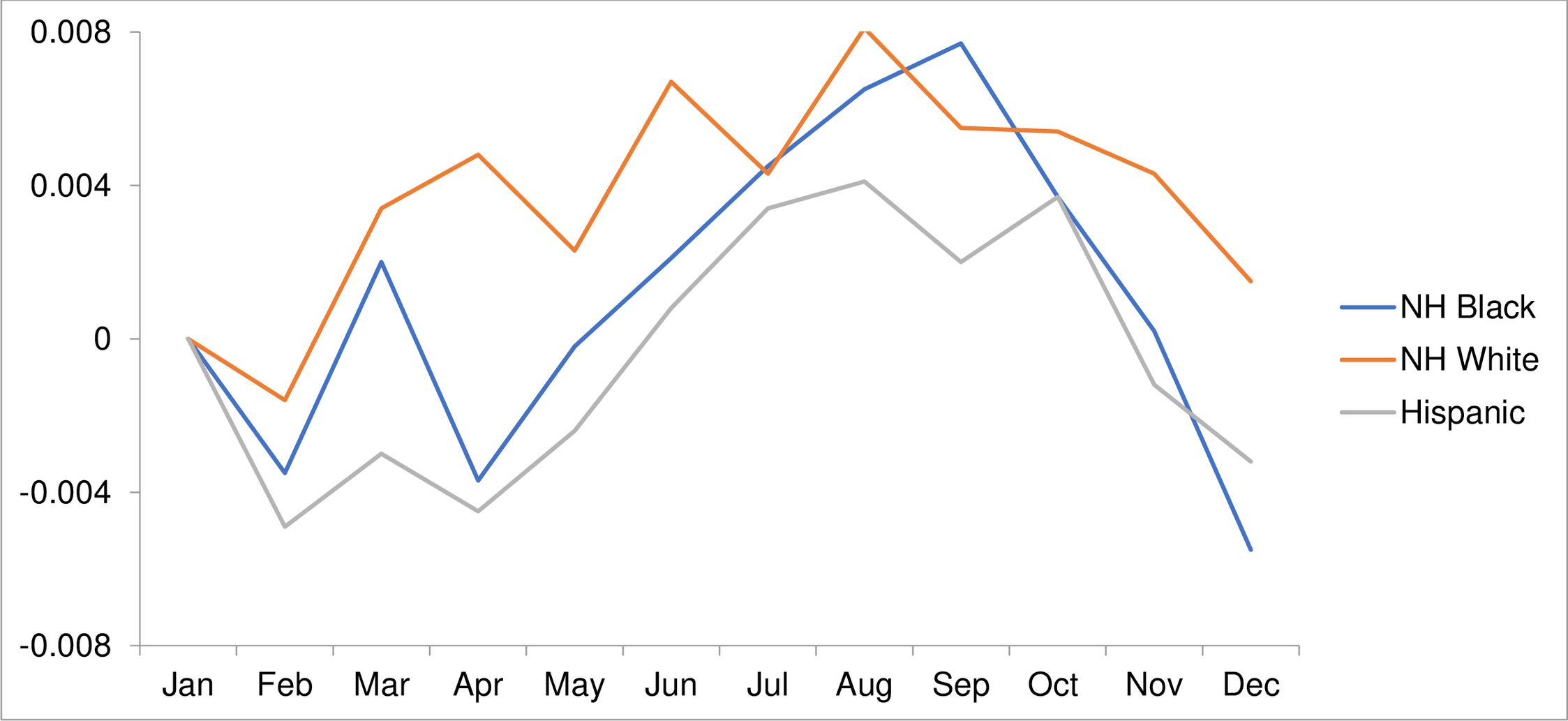

Supplement: S4 Fig — January fixed at 0. (TIF) [file pone.0295557.s004.tif]

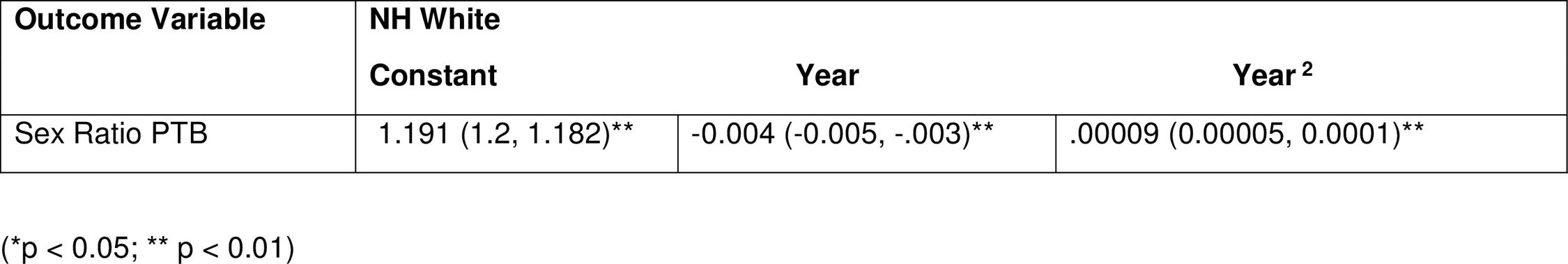

Supplement: S1 Table — (*p < 0.05; ** p < 0.01). (TIF) [file pone.0295557.s005.tif]

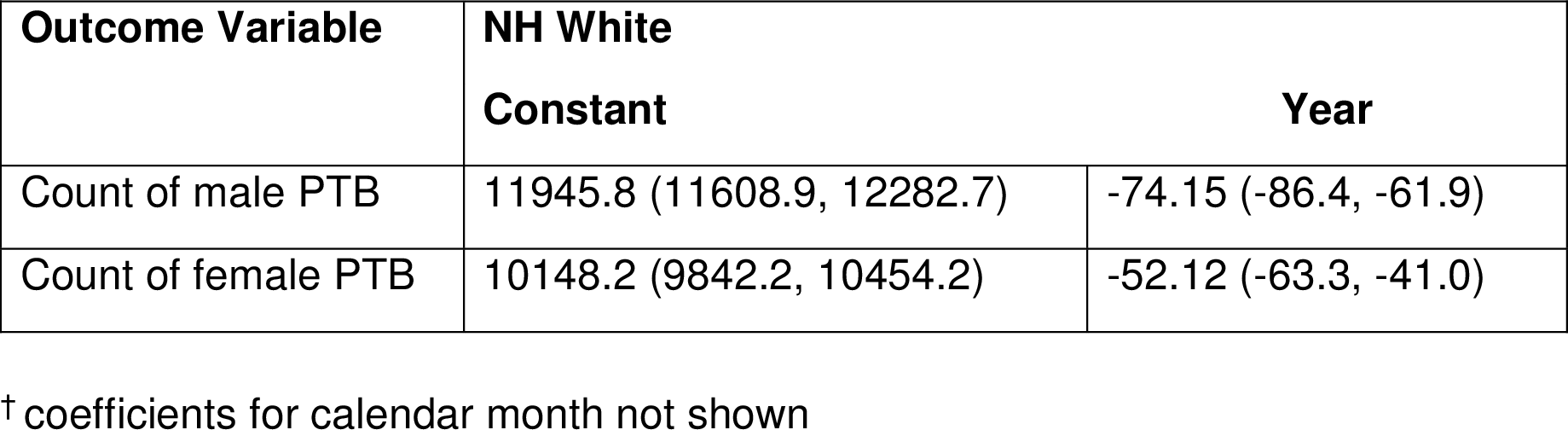

Supplement: S2 Table — † coefficients for calendar month not shown. (TIF) [file pone.0295557.s006.tif]

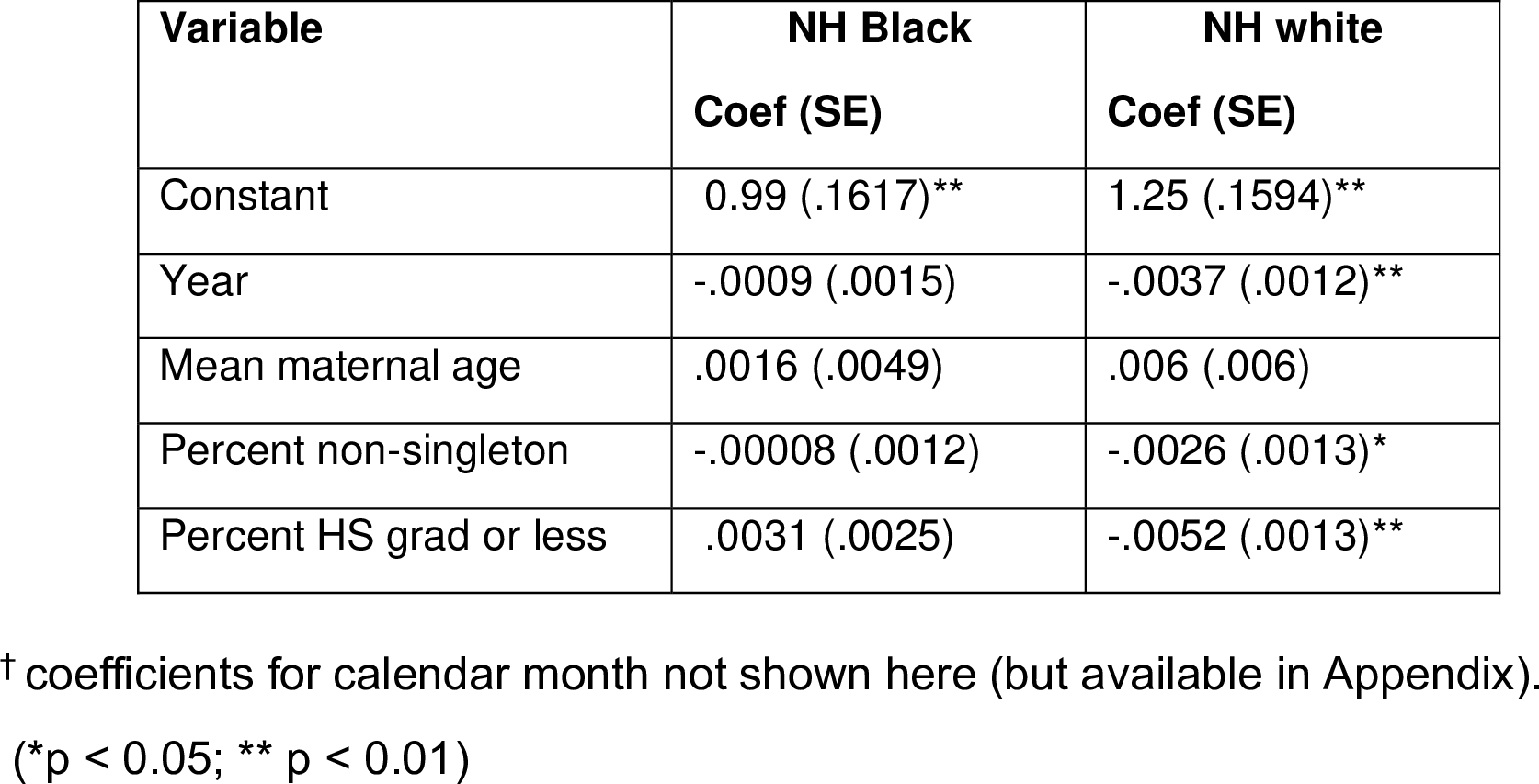

Supplement: S3 Table — † coefficients for calendar month not shown here (but available in the S3 Fig). (*p < 0.05; ** p < 0.01). (TIF) [file pone.0295557.s007.tif]

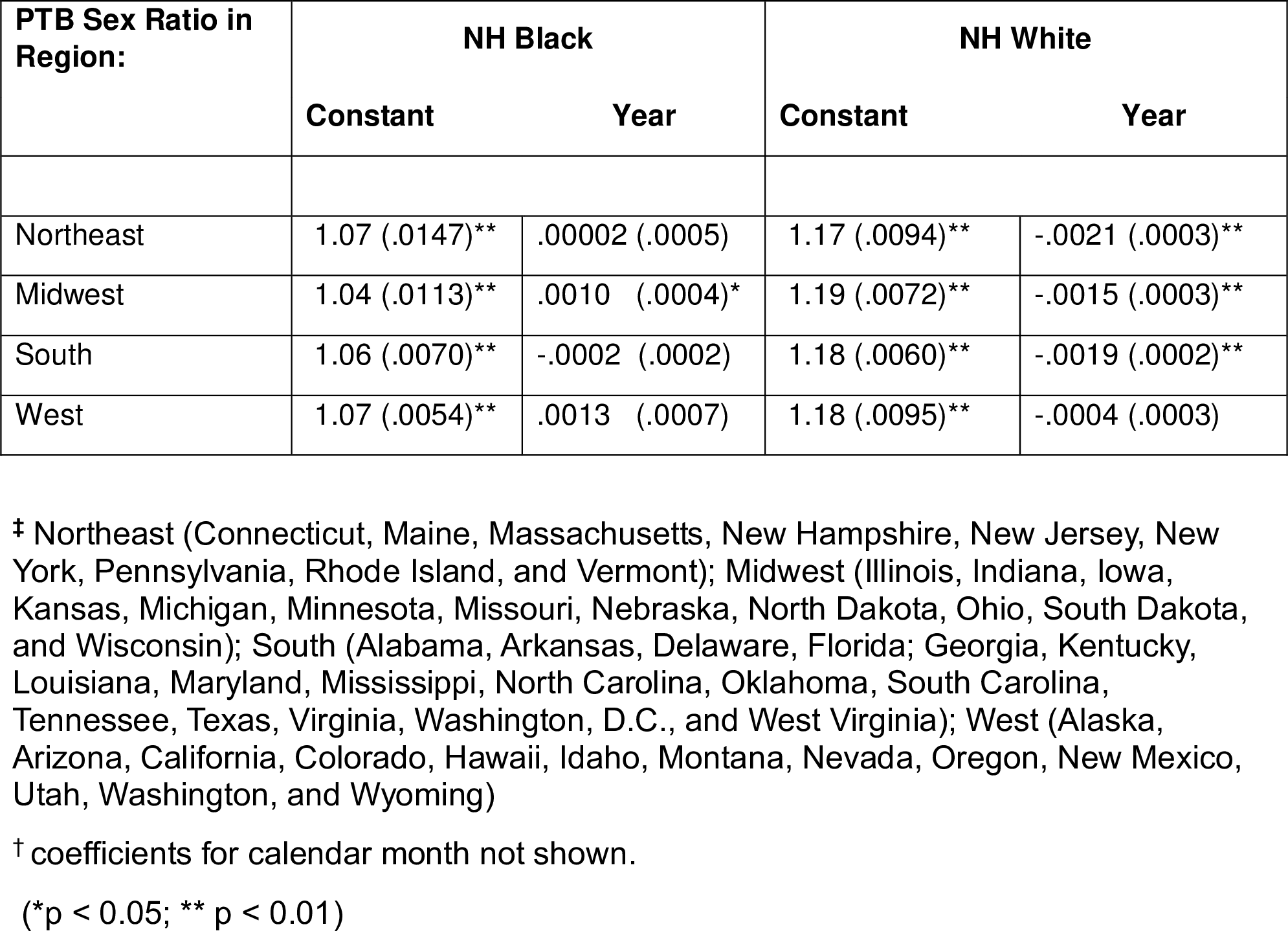

Supplement: S4 Table — † ‡ Northeast (Connecticut, Maine, Massachusetts, New Hampshire, New Jersey, New York, Pennsylvania, Rhode Island, and Vermont); Midwest (Illinois, Indiana, Iowa, Kansas, Michigan, Minnesota, Missouri, Nebraska, North Dakota, Ohio, South Dakota, and Wisconsin); South (Alabama, Arkansas, Delaware, Florida; Georgia, Kentucky, Louisiana, Maryland, Mississippi, North Carolina, Oklahoma, South Carolina, Tennessee, Texas, Virginia, Washington, D.C., and West Virginia); West (Alaska, Arizona, California, Colorado, Hawaii, Idaho, Montana, Nevada, Oregon, New Mexico, Utah, Washington, and Wyoming). † coefficients for calendar month not shown. (*p < 0.05; ** p < 0.01). (TIF) [file pone.0295557.s008.tif]
